# Supplementary material for: Moderate static magnetic fields prevent estrogen deficiency-induced bone loss: Evidence from ovariectomized mouse model and small sample size randomized controlled clinical trial
Source: PLoS One. 2025 Apr 29;20(4):e0314199. doi: 10.1371/journal.pone.0314199 (PMC12040201; doi:10.1371/journal.pone.0314199)
Supplement: S1 Data — (DOCX) [file pone.0314199.s001.docx]

**
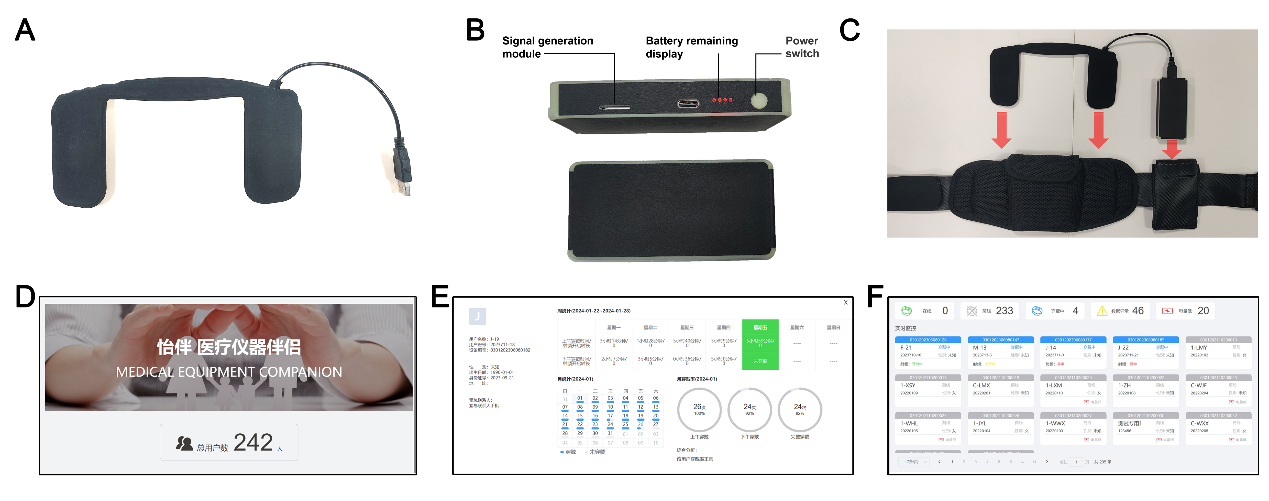
**

**Figure S1.  Wearable State Monitoring System of MMF device for clinical trial.** (**A**) Sensor. (**B**) Signal generation module with power supply. (**C**) Schematic diagram of the location of the monitoring system hardware components on MMF device. (**D**) Homepage of cloud-based monitoring system's backend software. (**E**) Real-time interface for displaying the wearing status of device. (**F**) Statistics screen for historical wear information.


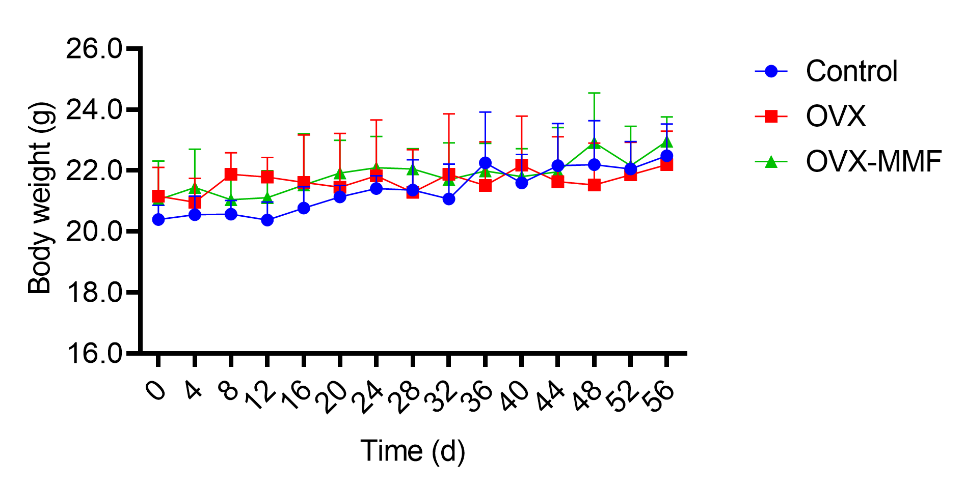


**Figure S2.  Effects of MMF exposure on body weight of mice.**


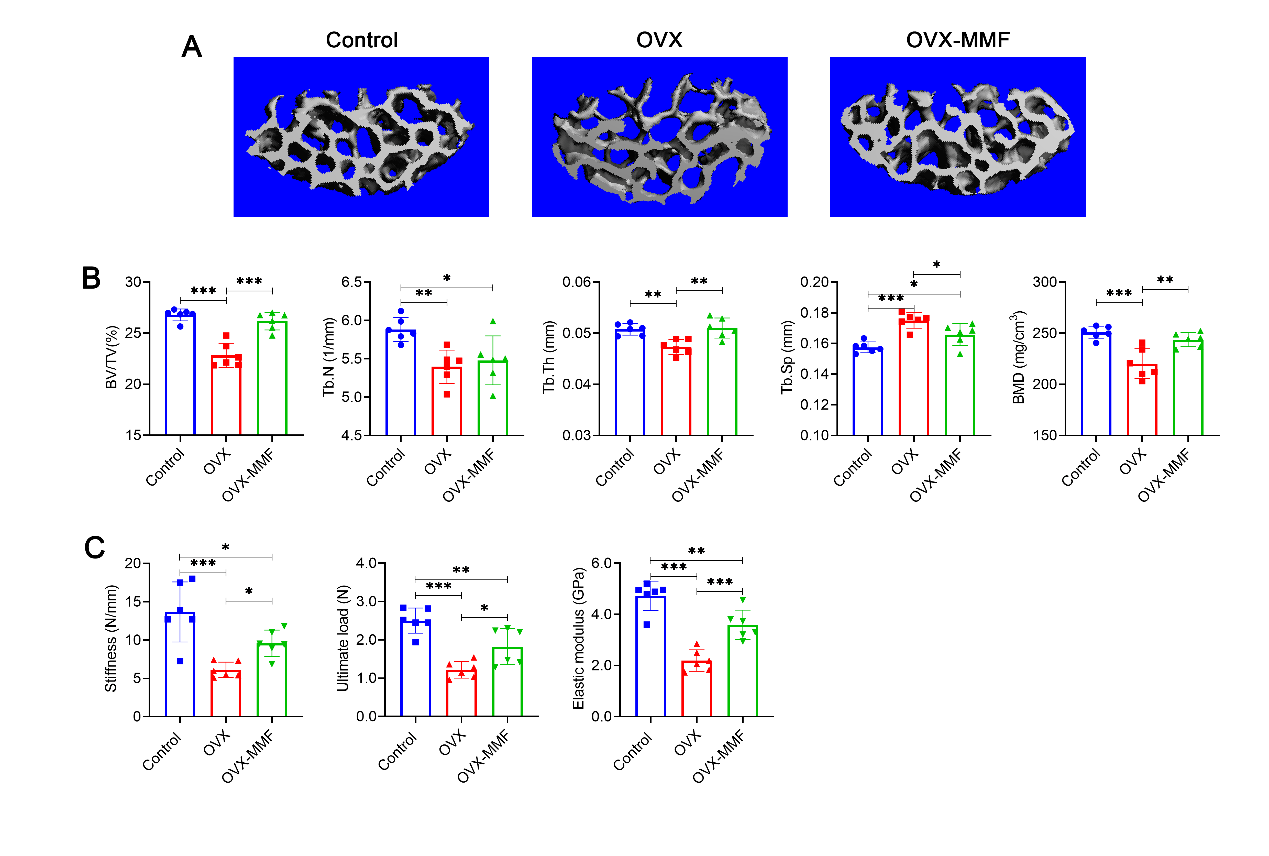


**Figure S3. Effects of MMF exposure on microstructure and mechanical properties of the lumbar vertebrae in OVX mice.** **(A)** Three-dimensional images of L4 lumbar vertebrae by micro-CT scanning. **(B)** Structural parameters of L4 trabecular bone, including BV/TV, Tb.N, Tb.Th, Tb.Sp, and BMD. **(C)** Mechanical properties of the L3 lumbar vertebrae in mice were detected through axial compression test, including stiffness, ultimate load, and elastic modulus. n =6. **P*<0.05, ***P*<0.01, ****P*<0.001.


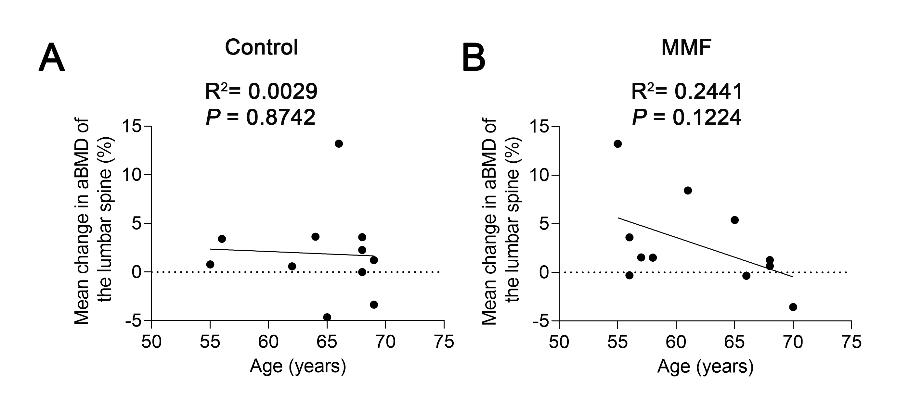


**Figure S4. The correlation of participants' BMD change rate and age after 90 days treatment in clinical trial.** (A) Control group. (B) MMF group.

Table S1. Bone mineral density (BMD) and bone mineral content (BMC) in mice was measured by DEXA.

|  | Control | OVX | OVX-MMF |
| --- | --- | --- | --- |
| **BMD (g/cm^3^)** |  |  |  |
| Whole body | 0.0374 ± 0.0057 | 0.0258 ± 0.0044** | 0.0321 ± 0.0050^#^ |
| Femur | 0.0228 ± 0.0037 | 0.0165 ± 0.0028** | 0.0201 ± 0.0026^#^ |
| Tibia | 0.0389 ± 0.0079 | 0.0306 ± 0.0039 | 0.0378 ± 0.0101 |
| Lumbar vertebrae | 0.0154 ± 0.0029 | 0.0102 ± 0.0019** | 0.0167 ± 0.0029^###^ |
| **BMC (g)** |  |  |  |
| Whole body | 0.6462 ± 0.1253 | 0.4839 ± 0.0822* | 0.5777 ± 0.1067 |
| Femur | 0.0221 ± 0.0027 | 0.0185 ± 0.0025 | 0.0235 ± 0.0039^#^ |
| Tibia | 0.0277 ± 0.0060 | 0.0224 ± 0.0025* | 0.0247 ± 0.0016 |
| Lumbar vertebrae | 0.0108 ± 0.0026 | 0.0068 ±0.0016* | 0.0121 ± 0.0032^##^ |

Data are shown in mean ± SD. n = 6. **P* < 0.05, ***P* < 0.01 *vs* Control; ^#^*P* < 0.05, ^##^*P* < 0.01, ^###^*P* < 0.001 *vs* OVX.

Table S2. Bone mineral density and bone turnover makers levels of participants post-treatment in clinical trial

|  | BASE treatment  /Control group  (n = 11) | MMF treatment  /MMF group  (n = 11) |
| --- | --- | --- |
| **Areal bone mineral density (g/cm²)** | | |
| Lumbar spine | 0.737 ± 0.102 | 0.764 ± 0.082 |
| Total hip | 0.709 ± 0.078 | 0.709 ± 0.079 |
| Femoral neck | 0.639 ± 0.076 | 0.639 ± 0.077 |
| **Bone turnover markers** |  |  |
| Serum OCN (μg/L) | 20.82 ± 6.29 | 15.99 ± 6.48 |
| Serum P1NP (μg/L) | 43.95± 23.72 | 49.01 ± 18.45 |
| Serum β-CTX (ng/L) | 346.9 ± 164.6 | 220.5 ± 178.6 |

Data are shown in mean ± SD. OCN = osteocalcin. P1NP=N-propeptide of type 1 procollagen. β-CTX = beta-isomer of the C-terminal telopeptide of type I collagen.
