# Supplementary material for: Moderate static magnetic fields prevent estrogen deficiency-induced bone loss: Evidence from ovariectomized mouse model and small sample size randomized controlled clinical trial
Source: PLoS One. 2025 Apr 29;20(4):e0314199. doi: 10.1371/journal.pone.0314199 (PMC12040201; doi:10.1371/journal.pone.0314199)
Supplement: S1 Protocol — (DOCX) [file pone.0314199.s003.docx]

**Title：**Investigation of the mechanisms of static magnetic fields' effects on bone remodeling based on dielectric properties and iron metabolism

## Integrity Statement

The research team hereby solemnly declares that this clinical trial will be conducted in accordance with the Declaration of Helsinki, the Ethical Review Measures for Biomedical Research Involving Humans issued by the National Health Commission, and the ethical principles outlined in China's Good Clinical Practice (GCP) guidelines. The study will follow the protocol approved by the Ethics Committee, with all trial data accurately recorded to ensure the scientific integrity of the research while safeguarding the health and rights of the participants. Furthermore, there are no conflicts of interest between the participating institutions or between the institutions and the study subjects.

## Funding Source

This study is funded by the National Natural Science Foundation of China (NSFC) Key Project (52037007) : “Investigation of the mechanisms of static magnetic fields' effects on bone remodeling based on dielectric properties and iron metabolism”.

## Research Procedure Execution Flowchart

| Activities/Assessments | Case Report (Yes/No) | -1 | 0 | T1 |
| --- | --- | --- | --- | --- |
|  |  | Screening | Baseline Variables Assessment/Assignment | Day 0 After Completion |
| Recruitment/Initial Screening | No | **×** |  |  |
| Questionnaire (Inclusion/Exclusion Criteria Form) | No | **×** |  |  |
| Eligibility Screening | Yes | **×** |  |  |
| Randomization | Yes |  | **×** |  |
| Magnetic Resonance Imaging (MRI) Examination | Yes | **×** |  | **×** |
| Area Bone Mineral Density (aBMD) Measurement | Yes |  | **×** | **×** |
| Blood routine | Yes |  | **×** | **×** |
| Bone turnover markers | Yes |  | **×** | **×** |
| Serum ferritin | Yes |  | **×** | **×** |
| VAS Score | Yes |  | **×** | **×** |
| Base treatment | Yes | **×** | **×** | **×** |
| MMF treatment | Yes |  |  | **×** |
| Data Collection | Yes |  | **×** | **×** |
| Data Evaluation/ Statistical analysis | No |  |  | **×** |

## Background

Osteoporosis has become an important health issue for people aged 50 and above in China, with middle-aged and elderly women experiencing particularly severe osteoporosis problems. In 2018, the Osteoporosis and Bone Mineral Diseases Branch of the Chinese Medical Association, in collaboration with the CDC, released an epidemiological survey on osteoporosis in China. The survey showed that the prevalence of osteoporosis in the 40-49 age group in China was 3.2%, with 2.2% in males and 4.3% in females. The incidence of osteoporosis in the population aged 50 and above is 19.2%, with 6.0% in males and 32.1% in females. The incidence of osteoporosis in the population aged 65 and above reaches 32.0%, with 10.7% in males and 51.6% in females. These data indicate that osteoporosis has become an important public health issue facing China, and the incidence of osteoporosis in women is significantly higher than that in men.

At present, various drugs for osteoporosis are constantly being developed, but many of them are not suitable for long-term use or have large adverse reactions, such as hormone drugs, which will increase the risk of uterine cancer, breast cancer, or nausea, vomiting and other adverse reactions. These unfavorable factors limit the scope of use of such drugs. Therefore, it is necessary to seek safer and more effective alternative treatment options.

Compared to drug therapy, physical therapy has better safety and fewer side effects. Pulsed electromagnetic fields have been approved for adjuvant therapy of clinical osteoporosis, and multiple clinical reports have shown that they have good therapeutic effects on osteoporosis. Compared to pulsed electromagnetic fields, static magnetic field has a more singular form of action and is the basis of the action of pulsed electromagnetic fields. The static magnetic field device and equipment used in this experiment have been proven to have good therapeutic effects on osteoporosis at the cellular and animal levels in vitro. This experiment intends to use a moderate static magnetic field device and wearable devices to intervene in postmenopausal women with lumbar osteoporosis, to investigate the intervention effect of medium intensity stable magnetic field on osteoporosis.

## Objectives

To study the effect of moderate static magnetic field on bone metabolism and iron metabolism in postmenopausal women with reduced lumbar bone density, and to evaluate the intervention effect of moderate static magnetic field on postmenopausal osteoporosis and its relationship with iron metabolism.

## Inclusion and exclusion Criteria

### Inclusion criteria

1. Female participants aged 55 to 70 years, with natural menopause.
2. Lumbar spine bone mineral density (BMD) T-score ≤ -2.5 as measured by dual-energy X-ray absorptiometry (DXA).
3. Participants who are fully informed, voluntarily sign the informed consent form, can communicate well either verbally or in writing, and demonstrate good compliance.

### Exclusion criteria

1. Prior spine surgery;
2. Had taken fracture within 6 months before enrolment;
3. Had taken Glucocorticoids, estrogen, or diuretic drugs within 6 months before enrolment;
4. Had taken drugs affecting bone metabolism within 6 months before enrolment, excluding calcium agents and ordinary vitamin D;
5. Other diseases that affect bone metabolism, including thyroid disease, osteomalacia, rheumatoid arthritis, tumor, cushing's disease, diabetes, etc.;
6. Uterus or ovaries removed;
7. Heart disease, or implantable pacemaker wearer;
8. BMI ≥ 28;
9. Hematological system diseases;
10. Abnormal liver and kidney function (Deviation of indicators by more than twice the normal value);
11. Alcoholism or drug abuse.

### Exit criteria

1. Withdrawal from treatment due to physical discomfort or other reasons.
2. Non-compliance with the prescribed treatment schedule.
3. Voluntary withdrawal by the participant.

## Trial design

A prospective randomized controlled study was conducted on postmenopausal women without osteoporotic lumbar compression fractures. According to the inclusion criteria, the included patients were randomly divided into a basic treatment group (control group, code A) and a moderate magnetic wearable device treatment group (experimental group, code B). All groups were given routine treatment, and the selected routine treatment method was salmon calcitonin nasal spray (trade name: Jinerli, Yingu Pharmaceutical, national drug approval number H20030905) 3.5 ml/month (20ug/day); Oral calcium carbonate D3 tablets (trade name: Caltech D, Wyeth Pharmaceuticals, national drug approval number H10950029) 2 tablets (600 mg)/day; Calcitriol (trade name: Luogaiquan, Roche Pharmaceuticals, national drug approval number J20150011) 0.5 ug/day.

Group A wears non-magnetic thoracolumbar braces for 6 hours a day, while Group B wears braces with moderate static magnetic fields (MMFs) for 6 hours a day. All patients in the group received 30 days of treatment as one course of treatment, for a total of one course of treatment. On the day of enrollment and 90 days after treatment, the subjects underwent bone mineral density (BMD), blood routine, bone metabolism, serum ferritin, and VAS evaluation. After 90 days of treatment, the subjects underwent tests such as blood routine, bone turnover markers, serum ferritin, and VAS evaluation. The technical flowchart is as follows:


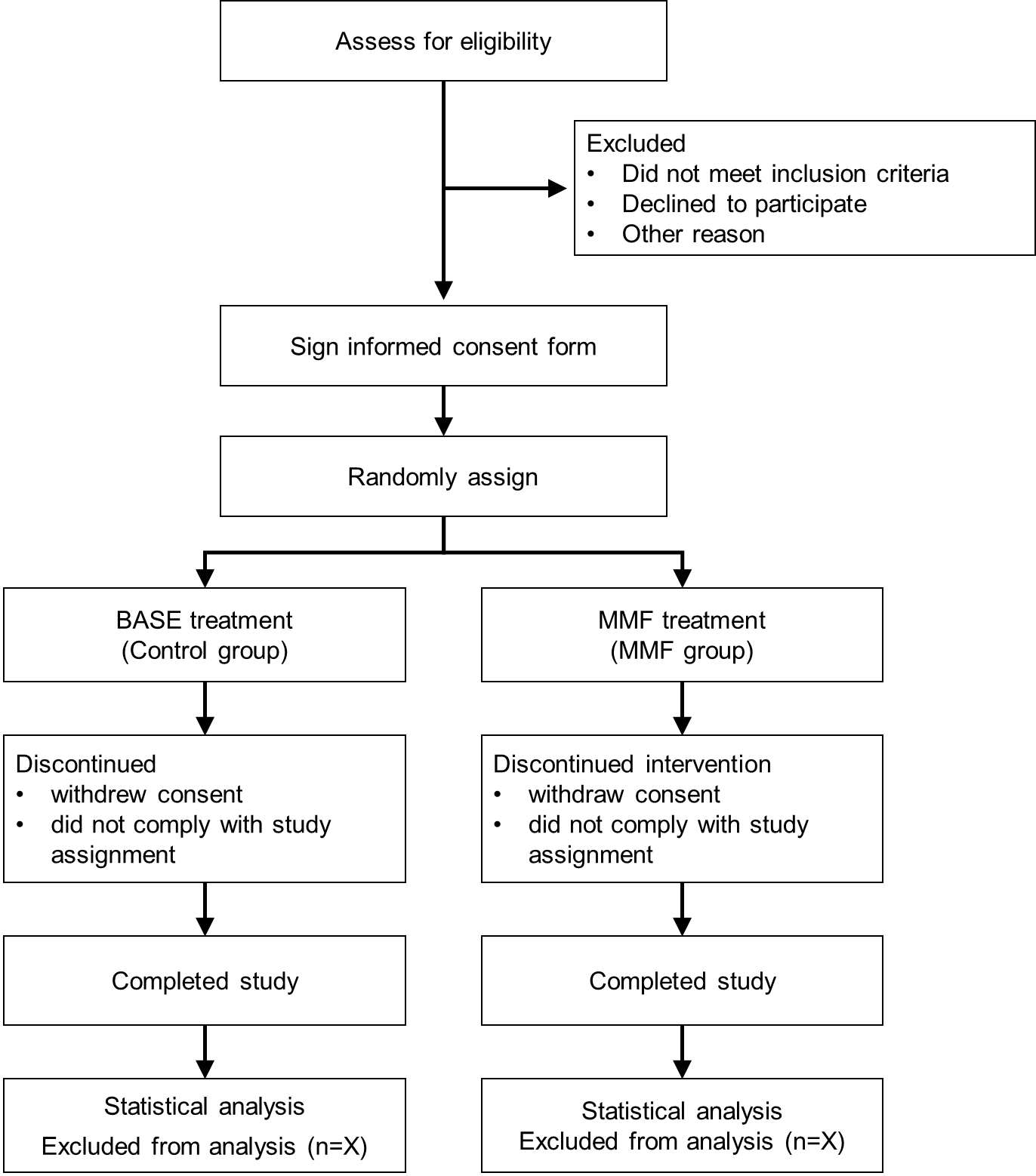


**Flowchart of the clinical trial.**

### Sample Size Calculation

Sample size was calculated (G*Power 3.1.9, Christian-Albrechts-Universitat, Kiel, Germany) using pain during areal bone mineral density (aBMD) as the primary outcome, α = 0.05, and power of 80% for between-group analysis. Considering a mean difference of 0.1 and a standard deviation of 0.07 in the mean aBMD between groups, sample size was estimated as 9 individuals per group. However, to account for losses during the follow-up period, the final sample size was 30 participants (15 per group).

### Randomisation

#### Sequence generation

Adopting the method of block random grouping.

#### Allocation concealment mechanism

A series of random numbers were generated on SPSS software with a seed number set by a statistician (who did not participate in the inclusion of cases and experimental research processes), where every 2 random numbers formed a block.

#### Implementation

The subjects were assigned to the corresponding groups in chronological order of enrollment. The grouping table is kept by the statistician and strictly confidential.

### Blinding

Blinding the subjects, testing physicians, data recorders, and data analysts throughout the entire trial process.

### Outcomes

#### Primary outcome

1. Areal bone mineral density (aBMD) of the lumbar spine, total hip, and femoral neck;
2. Visual Analogical Scale (VAS) of Low Back Pain
3. Serum ferritin

#### Secondary outcome

1. Bone turnover maker: Serum bone formation markers P1NP and OCN as well as the bone resorption marker β-CTX
2. Blood routine indicators

## Definition of Participant Eligibility

1. Right to Withdraw: Participants have the right to withdraw from the study at any stage for any reason. The researcher has an obligation to take necessary measures and respect the participant’s decision to exit the clinical trial.
2. Criteria for Participant Withdrawal by the Researcher: In the following circumstances, the researcher should actively consider allowing the participant to withdraw from the study:
   1. If the participant's condition worsens, such as developing fractures, and continued participation would be detrimental to their treatment.
   2. If the participant shows poor compliance.
   3. If adverse reactions intervene with the study protocol.
   4. If there are other circumstances that may increase the participant's risk or compromise the reliability of the study results.
3. Participant Voluntary Withdrawal: If a participant voluntarily withdraws from the clinical trial:
   1. They should not face any discrimination or retaliation, and their medical care should not be affected.
   2. They should be informed about alternative treatment options available to them.
   3. The researcher should make efforts to understand and document the reasons for the participant’s withdrawal in the original records.
4. Researcher Communication: The researcher must provide their contact information to the participant and actively obtain the participant's updated contact details to ensure timely follow-up.
5. Adverse Reaction Management: If a participant withdraws due to any adverse reactions, the researcher should take appropriate clinical measures based on the participant’s condition.
6. Follow-up After Adverse Event: If a participant withdraws due to an adverse event, the researcher should ensure follow-up monitoring until the adverse event is resolved.
7. Documentation of Withdrawal: All information regarding the participant’s withdrawal should be thoroughly recorded in the original documentation and periodically (e.g., in annual reports) submitted to the ethics committee.
8. Completion of Evaluations: After learning of a participant's withdrawal, the researcher should, if possible, complete all assessments and data collection that can still be carried out.
9. Retention of Data: Data collected prior to the participant’s withdrawal should be retained as part of the study database and should not be ignored or deleted.

## Definitions, Identification Methods, and Management of Adverse Events and Adverse Reactions

1. Collection of Safety Information: Researchers must actively collect safety information by engaging in thorough communication with participants, asking detailed questions, conducting comprehensive physical examinations, and reviewing laboratory test data. This information must be used to promptly and accurately assess any adverse events occurring during the study.
2. Adverse Event (AE): An adverse event is any unfavorable medical occurrence in a participant during the study that does not necessarily have a causal relationship with the treatment or intervention being studied. Adverse events may include unexpected symptoms, signs, or diseases, such as abnormal laboratory findings, that occur in association with the study intervention.
3. Serious Adverse Event (SAE): A serious adverse event refers to an adverse event that occurs during the study, either during the intervention or observation period, and involves any of the following:
4. Prolongation of hospitalization
5. Disability or impairment of function
6. Impact on work capacity
7. Life-threatening events
8. Death
9. Congenital malformations
10. Assessment of Adverse Events: After identifying an adverse event, the researcher must first determine if it qualifies as a serious adverse event. Ordinary adverse events should be handled clinically according to their nature and documented in the CRF (Case Report Form) under the adverse event record.
11. Reporting of Adverse Reactions: If an adverse event is clearly identified as a drug-related adverse reaction, it should be reported according to the center’s adverse reaction reporting procedure.
12. Management of Serious Adverse Events:
13. If a serious adverse event threatens the participant's life, immediate and appropriate medical treatment should be provided. The participant's safety should be prioritized, and emotional distress should be addressed.
14. The serious adverse event should be reported to the center's principal investigator and the ethics committee within 12 hours of occurrence.
15. Effective communication with the participant and their family should be maintained throughout the process.
16. Follow-up of Adverse Events: All adverse events should be followed up to assess their outcome. Appropriate actions should be taken to resolve the issue, or the participant’s condition should be stabilized.

## Participant Recruitment

Participants will be recruited from those attending the outpatient clinics or undergoing physical examinations at People's Hospital of Longhua, who have been diagnosed with postmenopausal osteoporosis. The researcher will assess each potential participant based on the inclusion criteria specified for this clinical trial to determine their eligibility. If eligible, the researcher will provide detailed information about the study to the participant and ascertain their willingness to participate. For initially recruited participants, further screening will be conducted based on the exclusion criteria outlined in this study to confirm their suitability for inclusion in the clinical trial.

## Statistical methods

The normally distributed continuous data will be expressed as mean ± standard deviation (SD), as median (IQR) for endpoints without a normal distribution. The data about mean aBMD percentage change in clinical will be expressed trial as mean ± standard error (SE), as per the reporting conventions for this study area. The normal distribution was tested by Kolmogorov-Smirnov test with *P*>0.10. The comparison of group differences in normally distributed continuous data was performed using t-tests. Non-parametric rank-based tests were employed for the comparison of group differences in non-normally distributed continuous data. *P*<0.05 will be considered to be statistically significant. All the statistical data will be analyzed by GraphPad Prism (version 8; GraphPad Software, Inc., San Diego, CA, USA).
